# Potential Target Discovery and Drug Repurposing for Coronaviruses: Study Involving a Knowledge Graph–Based Approach

**DOI:** 10.2196/45225

**Published:** 2023-10-20

**Authors:** Pei Lou, An Fang, Wanqing Zhao, Kuanda Yao, Yusheng Yang, Jiahui Hu

**Affiliations:** 1 Institute of Medical Information Chinese Academy of Medical Sciences & Peking Union Medical College Beijing China

**Keywords:** coronavirus, heterogeneous data integration, knowledge graph embedding, drug repurposing, interpretable prediction, COVID-19

## Abstract

**Background:**

The global pandemics of severe acute respiratory syndrome, Middle East respiratory syndrome, and COVID-19 have caused unprecedented crises for public health. Coronaviruses are constantly evolving, and it is unknown which new coronavirus will emerge and when the next coronavirus will sweep across the world. Knowledge graphs are expected to help discover the pathogenicity and transmission mechanism of viruses.

**Objective:**

The aim of this study was to discover potential targets and candidate drugs to repurpose for coronaviruses through a knowledge graph–based approach.

**Methods:**

We propose a computational and evidence-based knowledge discovery approach to identify potential targets and candidate drugs for coronaviruses from biomedical literature and well-known knowledge bases. To organize the semantic triples extracted automatically from biomedical literature, a semantic conversion model was designed. The literature knowledge was associated and integrated with existing drug and gene knowledge through semantic mapping, and the coronavirus knowledge graph (CovKG) was constructed. We adopted both the knowledge graph embedding model and the semantic reasoning mechanism to discover unrecorded mechanisms of drug action as well as potential targets and drug candidates. Furthermore, we have provided evidence-based support with a scoring and backtracking mechanism.

**Results:**

The constructed CovKG contains 17,369,620 triples, of which 641,195 were extracted from biomedical literature, covering 13,065 concept unique identifiers, 209 semantic types, and 97 semantic relations of the Unified Medical Language System. Through multi-source knowledge integration, 475 drugs and 262 targets were mapped to existing knowledge, and 41 new drug mechanisms of action were found by semantic reasoning, which were not recorded in the existing knowledge base. Among the knowledge graph embedding models, TransR outperformed others (mean reciprocal rank=0.2510, Hits@10=0.3505). A total of 33 potential targets and 18 drug candidates were identified for coronaviruses. Among them, 7 novel drugs (ie, quinine, nelfinavir, ivermectin, asunaprevir, tylophorine, *Artemisia annua* extract, and resveratrol) and 3 highly ranked targets (ie, angiotensin converting enzyme 2, transmembrane serine protease 2, and M protein) were further discussed.

**Conclusions:**

We showed the effectiveness of a knowledge graph–based approach in potential target discovery and drug repurposing for coronaviruses. Our approach can be extended to other viruses or diseases for biomedical knowledge discovery and relevant applications.

## Introduction

The global COVID-19 pandemic has heavily burdened the normal life and work of human beings, and has caused an unprecedented crisis in medical care, and social and economic development [1-3]. Although the impact of COVID-19 is unprecedented, it is not the first outbreak of a coronavirus in humans.

Coronaviruses represent a diverse group of enveloped viruses with crown-like spikes on the surface, which were first discovered in the 1930s, and human coronaviruses were first identified in the 1960s [4]. To date, 7 coronaviruses are known to infect humans. In addition to the common human coronaviruses (229E, NL63, OC43, and HKU1), severe acute respiratory syndrome coronavirus (SARS-CoV), Middle East respiratory syndrome coronavirus (MERS-CoV), and SARS-CoV-2 are highly pathogenic, causing a high number of deaths and global panic in this century [5,6]. Currently, there are no reports of clinically effective prevention or treatment strategies for coronavirus infection. More seriously, the continued emergence of novel coronaviruses and their zoonotic potential further exacerbate concerns about the future of public health [7].

Coronaviruses are still evolving, and it is unknown how and when mutated strains of coronaviruses emerge [8,9]. Despite ongoing progress in vaccination against coronaviruses and mass antiviral screening being undertaken rapidly, there is uncertainty about the protection that existing measures may provide [10]. Targets, drugs, and mechanisms of drug action for coronaviruses urgently need to be discovered.

However, new drug development usually takes about 15 years. In the early stages of drug development, tens of thousands of active molecules need to be tested, but only 1 of them may end up as a drug on the market [10-12]. Since traditional drug target discovery processes are time-consuming and infeasible, computational methods can be considered as some of the promising avenues to improve the speed and efficiency of drug target discovery.

The biomedical literature has abundant information on the molecular virology of coronaviruses [13]. Existing drug and genetic knowledge bases already contain a wealth of proven biomedical knowledge. It is advisable to extract coronavirus-related information from vast biomedical literature and integrate it with existing knowledge to discover potential targets and drug candidates to repurpose for coronaviruses.

Recently, a knowledge discovery approach combining literature-based discovery and a knowledge graph (KG) has emerged and is promising for drug repurposing. Literature-based discovery is a mature method widely used to automatically mine potential knowledge associations from the literature [14]. A KG is a graph computing method that describes concepts and their relationships in the physical world in a symbolic form [15,16]. With the development of various KG embedding methods, large-scale KGs can be used for data mining and knowledge discovery [17-20]. For example, the Rephetio project constructed an integrative network, Hetionet, by integrating data from 29 public biomedical resources [21]. The drug repurposing KG (DRKG) [22] integrated Hetionet with DrugBank, GNBR, String, IntAct, and DGIdb, consisting of nearly 100,000 entities and 6 million relationships, which was more than twice the size of Hetionet. Although these large-scale databases have integrated rich biomedical knowledge, the data fitting effect for the required semantic types in specific real-world use cases based on them may be ineffective, since the computational model considers all semantic types in the graph simultaneously. To improve the performance and computational efficiency for specific tasks, a KG filtering method considering the trade-off between data quantity and quality was proposed and applied in the prediction task of drug repurposing [21]. The current progress of integrating heterogeneous knowledge is relatively slow [23,24]. Other than integrating heterogeneous data, a notable drug repurposing approach [25] was developed via KG completion based only on literature knowledge, and a specific pattern was defined to filter out the relationship between drugs and COVID-19.

Existing efforts have explored computational methods from different perspectives for drug repurposing. However, available KGs were only used for a single coronavirus, especially SARS-CoV-2. The clinical manifestations of coronavirus infection are similar. Patients infected with SARS-CoV, MERS-CoV, and SARS-CoV-2 all exhibit flu-like symptoms and may progress to pneumonia and dyspnea with acute respiratory distress syndrome and multi-organ failure in severe cases [26]. Coronaviruses, especially human coronaviruses, have many similarities at the molecular level [27]. The analysis data of the gene sequences of the coronavirus spike suggest that coronaviruses have a similar evolution [28]. Constructing specialized KGs for all identified coronaviruses is beneficial to uncover underlying biological knowledge among various coronaviruses. Although some biological studies have attempted to explore the connections and differences among various coronaviruses, no KGs for various coronaviruses have been reported.

This study aimed to explore potential targets and drug candidates to repurpose for coronaviruses, using KGs, to benefit the prevention and treatment of highly pathogenic coronavirus infections. The novelty of this work is taking the initiative to employ KGs for various coronaviruses rather than a single coronavirus. This work has the following main contributions: (1) considering computational cost and efficiency, coronavirus-related data from the literature were initially filtered and semantic information was automatically extracted from a large-scale biomedical literature database; (2) a coronavirus KG (CovKG) was constructed with the integration of heterogeneous data from both the biomedical literature and structured drug and gene databases; (3) 6 state-of-the-art KG embedding methods were evaluated for drug repurposing of coronaviruses; and (4) the unrecorded mechanisms of drug action as well as potential targets and drug candidates were discovered and discussed with evidence-based support, which further enhanced the interpretability of the predicted results.

## Methods

### Overview

**Figure 1:**
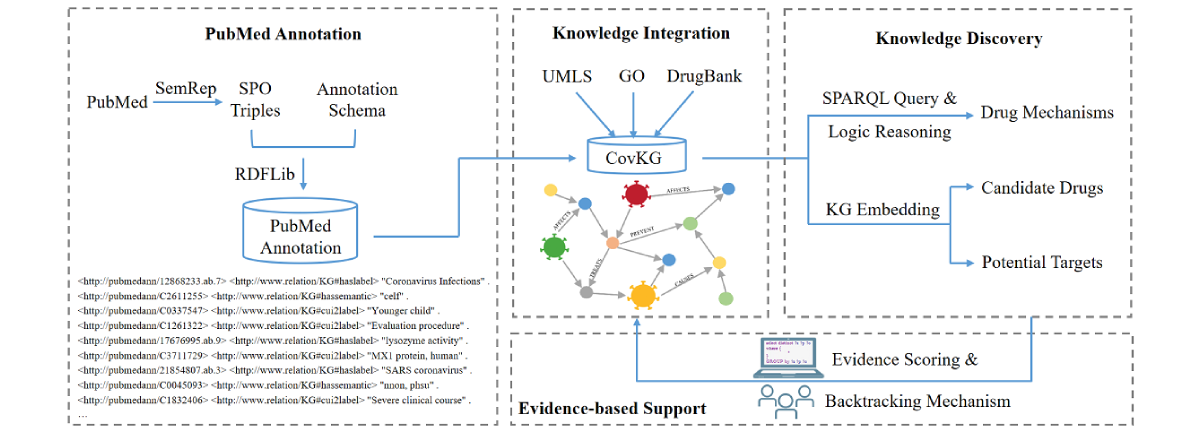
Diagram illustrating the workflow of our approach. CovKG: coronavirus knowledge graph; GO: Gene Ontology; KG: knowledge graph; SPO: subject- predicate-object; UMLS: Unified Medical Language System.

We first describe our data sources, including the biomedical literature data source, drug and target knowledge source, and gene product knowledge source. Then, we construct the CovKG by extracting information from biomedical literature and associating it with the Unified Medical Language System (UMLS), DrugBank, and Gene Ontology (GO) in a unified format. Next, 6 KG embedding models are explored to predict potential targets and candidate drugs to repurpose for coronaviruses, and the semantic reasoning technique of KGs is employed to find the unrecorded mechanisms of drug action in the existing drug knowledge base. Furthermore, evidence-based supports are provided with a scoring and backtracking mechanism. The workflow diagram illustrating our approach is shown in Figure 1.

### Data Collection

PubMed contains more than 34 million biomedical articles, and the data volume grows by thousands of articles every day [29]. It is one of the most commonly used literature resources. In this study, PubMed was considered as the basic data source for biomedical literature knowledge on coronaviruses. To explore the potential relationships among coronavirus-related diseases, targets, and drugs, we used “coronavirus” as the search term in PubMed to obtain relevant biomedical literature data. Research literature published from January 2000 to June 2022 was collected. Then, a total of 18,687 most relevant publications were obtained after deduplication, from which we extracted coronavirus-related semantic relations.

DrugBank is a comprehensive database on drugs and targets [30]. It consists of 15,305,066 triples, containing 13,580 drug concepts and 84 relations, such as drug interaction, category, dosage, mixture, and pathway. As a bioinformatics and cheminformatics resource, it records detailed drug and target information. In this study, we adopted DrugBank data in Resource Description Framework (RDF) format.

GO is the largest source of information on the functions of gene products [31]. It has a total of 1,423,359 triples, containing 47,345 gene concepts and 49 relations, such as label, subclass, and definition. The GO data are provided in Web Ontology Language (OWL) format.

### CovKG Construction

#### Biomedical Literature Knowledge Base

The biomedical literature contains abundant information related to coronaviruses and is an important knowledge source for the construction of the CovKG. Therefore, we first extracted the triples containing biomedical knowledge based on PubMed literature data and then designed a semantic conversion model to organize the knowledge in an orderly manner. Finally, we stored the biomedical literature knowledge through a unified standard. The biomedical literature knowledge base obtained has been named PubMedAnn.

##### Automatic Biomedical Information Extraction

**Figure 2:**
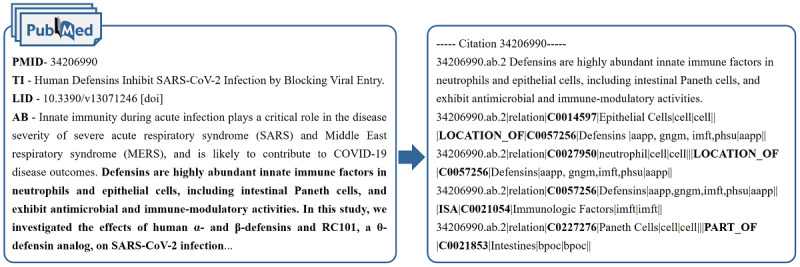
An example of automatic biomedical information extraction using SemRep. The bold content on the right represents the triplets extracted from the text on the left, denoted as “CUI-Relation-CUI”. AB: Abstract; CUI: concept unique identifier; LID: Location ID; PMID: PubMed ID; TI: Title.

SemRep is an effective off-the-shelf tool for identifying entities and relations from text and linking them to UMLS standard terminology [32]. We first leveraged SemRep to automatically extract semantic triples from the retrieved literature. Figure 2 provides an example of automatic biomedical information extraction using SemRep. In Figure 2, one or more semantic types are assigned to each identified entity, for example, the label and semantic type corresponding to “C0014597” are “Epithelial Cells” and “Cell,” respectively. The title and abstract of each article are segmented by sentences, and each sentence may contain one or more triples. On the left side of Figure 2, 4 triples are extracted from the second sentence of the article abstract (34206990.ab.2) with PubMed ID (PMID) 34206990, as shown on the right side of Figure 2. Entities in each triple were identified and normalized to concept unique identifiers (CUIs) in the UMLS Metathesaurus [33], and relations were linked to the semantic relations in the UMLS Semantic Network [34].

##### Semantic Structure Conversion Model

**Figure 3:**
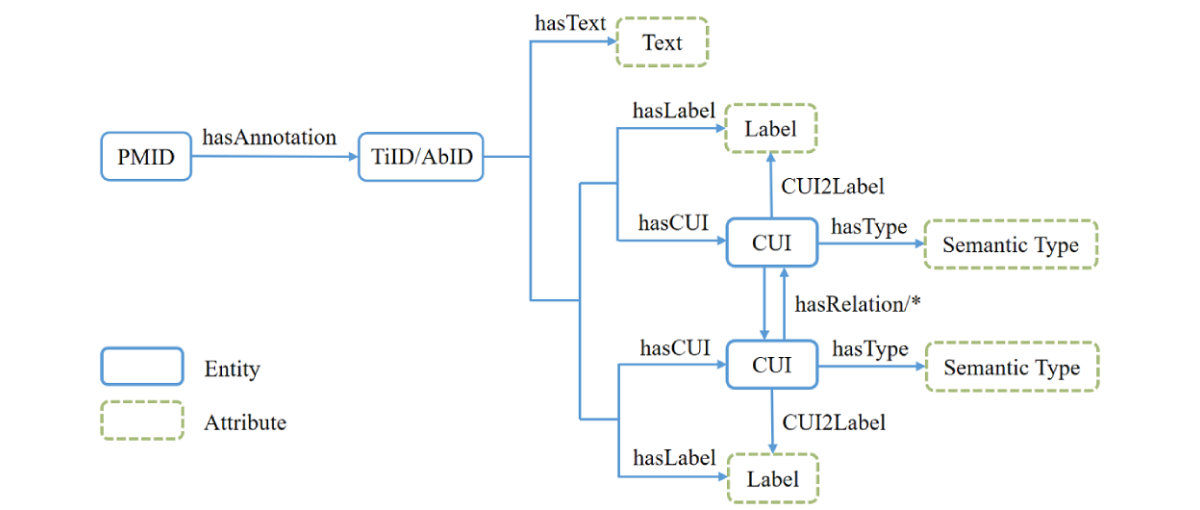
Semantic structure of PubMedAnn. Entities and attributes are distinguished by different colors and different types of borders. AbID: abstract ID; CUI: concept unique identifier; PMID: PubMed ID; TiID: title ID.

For biomedical literature processed by SemRep, there are title annotations and abstract annotations. These annotations correspondingly have their own text content. Additionally, entities identified by SemRep in these texts have their own CUIs and labels. Furthermore, various relationships exist among CUIs. Then, to organize the coronavirus-related information of biomedical literature, a semantic structure conversion model was defined, as shown in Figure 3.

In the conversion model, PMID represents the ID of a coronavirus-related article retrieved from PubMed. The title ID (TiID) and the abstract ID (AbID) of the literature are associated with the PMID through the relationship of “hasAnnotation.” Similarly, relationships, such as “hasText,” “hasCUI,” and “hasLabel,” have been defined. The relations between CUIs extracted by SemRep are represented by “hasRelation/*,” where “*” indicates the relation type of the corresponding triple. Moreover, “CUI2Label” and “hasType” have been defined for the relations between CUI and its label, and between CUI and its semantic type, respectively.

In the semantic structure of PubMedAnn, the semantic relationship between biomedical entities is designed and the sources of semantic type are clearly recorded through PMID, TiID, and AbID, providing an interpretable path for the subsequent model predictions.

##### Standardized Storage of Graph Data

RDF is a standard model for data interaction, where node types include Uniform Resource Identifier references, blank nodes, and literals [35]. In this study, we employed RDFLib [36] to unify the data format and convert the data extracted by SemRep into RDF.

Thus, according to the designed semantic structure conversion model, we extracted semantic triples from the data processed by SemRep and then used RDFLib to convert them into the RDF format required for KG construction. The transformed data were stored in a graph database (PubMedAnn).

#### Multi-Source Knowledge Integration

**Figure 4:**
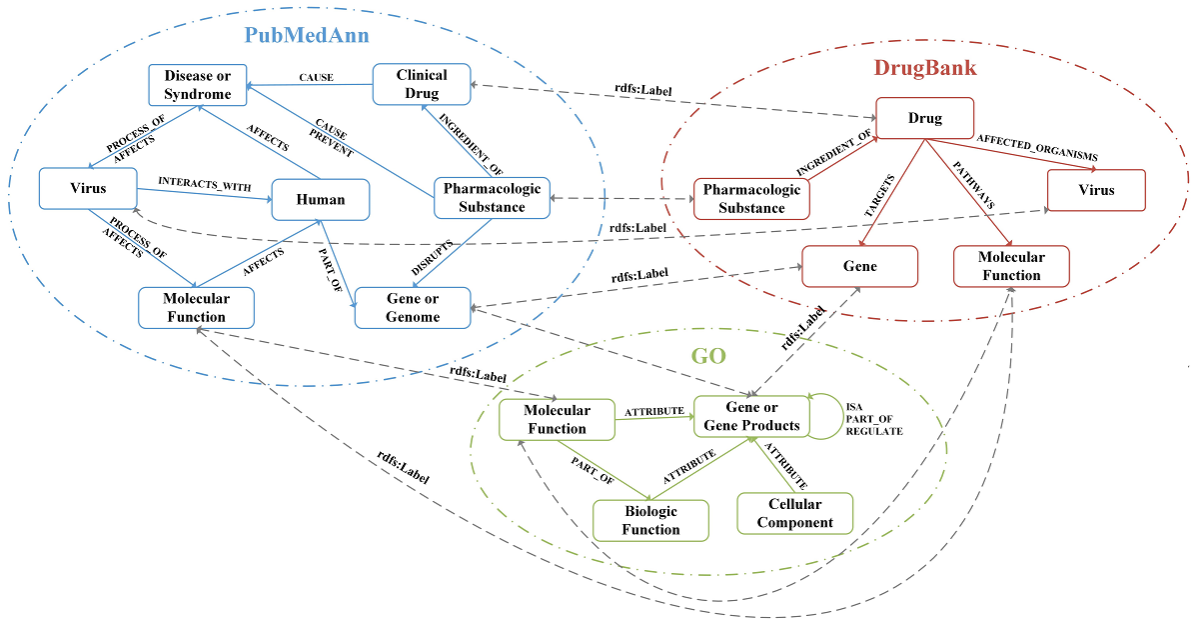
The main schema of the CovKG with the integration of multi-source biomedical knowledge from PubMedAnn, DrugBank, and GO. In the CovKG, nodes represent semantic types and edges represent relations between semantic types. CovKG: coronavirus knowledge graph; GO: Gene Ontology.

To discover potential targets and candidate drugs to repurpose for coronaviruses, the CovKG was constructed by integrating multi-source biomedical knowledge from the PubMedAnn, DrugBank, and GO databases. This study focused on the relationships between semantic types closely related to coronaviruses, including virus, disease, drug, gene, host, etc. Figure 4 shows the main schema of the CovKG.

Regarding the specific integration process across heterogeneous knowledge bases, the semantic types in PubMedAnn were mapped to those already existing in DrugBank and GO, for example, “Clinical drug,” “Virus,” and “Molecular Function” can be mapped to DrugBank, and “Gene or genome” and “Molecular Function” can be mapped to GO. Meanwhile, the relations were integrated by adding the relations of “AFFECTED_ORGANISMS,” “PATHWAYS,” and “TARGETS” in DrugBank and the relations of “ISA,” “PART_OF,” and “REGULATED” in GO to the schema of the CovKG. The attribute “rdfs:Label” was used to associate entities with the same semantic type in different knowledge bases.

In view of the excellent performance of GraphDB [37] in the field of graph databases, we used it to store the CovKG, and implemented knowledge query and logic reasoning based on the SPARQL language.

### Knowledge Discovery

#### KG Embedding Models

Knowledge representation learning is mainly oriented to entities and relations in KGs, and aims to represent the semantic information of research objects as dense low-dimensional real-valued vectors. We explored 2 classes of KG embedding methods: TransE [38], TransR [39], and RotatE [40] for translational models, and RESCAL [41], DistMult [42], and ComplEx [43] for semantic matching models. These 6 models were used to learn vector representations of entities and relations for subsequent knowledge discovery.

TransE is a representative translational distance model that represents entities and relations as vectors in the same semantic space of dimension. TransE extracts vectors from the head entity and relation, and performs L1 norm or L2 norm operations to make the obtained results approximate to the vectors in the tail entity. TransE is simple and has good prediction performance. However, TransE only models 1-1 relations and fails to embed 1-n and n-n relations. To solve this problem, several other solutions have been proposed, including TransR and RotatE.

TransR separates the relation space from the entity space, and semantic spaces do not need to have the same dimensions. Each of these spaces captures a different aspect of the head entity *h* or the tail entity *t*, which is related to a distinct relationship *r*. Similar to TransE, the score function *f_r_* of TransR measures the Euclidean distance between *h*+*r* and *t*, and *f_r_* is as follows:



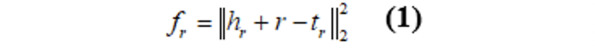



RotatE maps entities and relations to a complex vector space and defines each relation as a rotation from the head entity to the tail entity. RotatE uses a self-adversarial negative sampling method that samples negative triples based on the current embedding model. The score function *d_r_* (*h*,*t*) of RotatE measures the angular distance between the head and tail elements, and *d_r_* (*h*,*t*) is as follows:



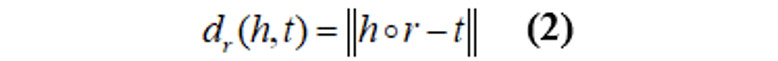



RESCAL is the most basic model based on tensor decomposition. The core idea of RESCAL is to encode the entire KG into a 3D tensor, and decompose a core tensor and a factor matrix from it. Each slice *X_k_* of the 2D matrix represents a tensor representation of a relation in the core tensor, *k* denotes the number of relationships, and *A* represents the entity set matrix. The result restored by the core tensor and factor matrix is regarded as the probability of the corresponding triple. If the probability is greater than a certain threshold, the corresponding triple is correct. The score function *f_r_* (*h*,*t*) for *h*, *t* ϵ *R^d^*, captures pairwise interactions between entities in *h* and *t* through relationship matrix *M_r_* that is the collection of all individual *R_k_* matrices and is of dimension *d*×*d*, and the formulas are as follows:



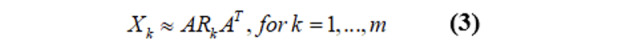





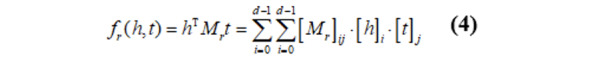



DistMult alleviates the overfitting problem by reducing the number of parameters. DistMult simplifies RESCAL by restricting *M_r_* from a general asymmetric *r*×*r* matrix to a diagonal square matrix, thus reducing the number of parameters per relation. DistMult introduces vector embedding *r* ϵ *R^d^*, and then, the score function is as follows:



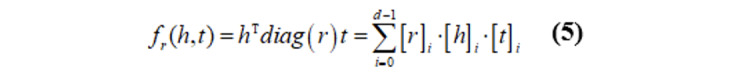



ComplEx extends DistMult by introducing complex-valued embedding to better model asymmetric relations. In ComplEx, the embedding of entity and relation no longer exists in the real vector space but in the complex vector space. An essential strategy is to compute joint representations for the entities, regardless of their roles as subjects or objects, and perform dot products on these embeddings, with a score function as follows:



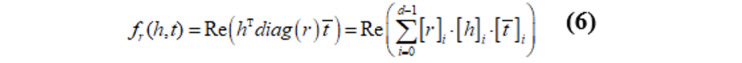



#### Link Prediction and Similarity Calculation

**Figure 5:**
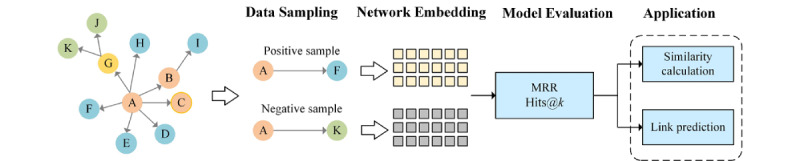
Knowledge discovery process based on network representation learning. MRR: mean reciprocal rank.

In the context of biomedical networks, KG embedding helps to discover previously unknown associations or interactions. Here, we employed the KG embedding model to obtain low-dimensional vector representations, which were further used for link prediction and similarity calculation to find potential targets and drug candidates for coronaviruses, respectively. The knowledge discovery process based on network representation learning is shown in Figure 5.

Link prediction is the task of predicting the possibility of an unknown connection between 2 entities in a given network through known information [44]. In this task, the existing triples in the graph are used as positive samples, while the unknown entities or relations are negatively sampled.

Through similarity calculation, the predicted entity should be most similar to the recorded drug entity in the graph. The embedding of the recorded drug entity was denoted as *e*, and the embedding of the predicted drug entity was denoted as *e′*. Cosine similarity [45] was used to calculate the similarity between entity pairs. Since 6 KG embedding models were employed for KG completion in our study, the similarity calculation formula was as follows:



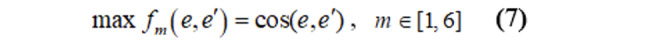



#### Semantic Reasoning–Based Knowledge Discovery

Based on the CovKG integrating multi-source biomedical knowledge (ie, PubMedAnn, DrugBank, and GO), we designed a semantic reasoning-based knowledge discovery pattern to identify unrecorded mechanisms of drug action. SPARQL was used to filter triples with the semantic type “head entity (drug) - r - tail entity (target)” in PubMedAnn, where the head entity was mapped to the drug name in DrugBank and the tail entity was mapped to the target name in GO. Thus, all drug-target relationships recorded in PubMedAnn were obtained, which were then compared with the existing drug targets in the authoritative database to find the unrecorded mechanisms of drug action.

### Evaluation Criteria

We conducted cross-validation to evaluate the model performance for drug and target predictions. All triples were randomly shuffled 5 times and split into a training set (70%) and a test set (30%) with the same ratios. The mean reciprocal rank (MRR) and Hits@*k*, which have been widely used as evaluation metrics [46], were adopted to measure the performance of our KG embedding models.

The MRR is the average inverse rank of all test triples and is calculated as follows:



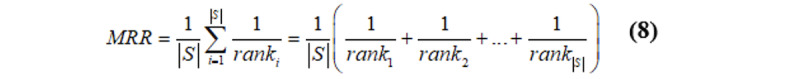



Hits@*k* measures the percentage of true triples appearing in the top *k* ranked triples [47]. Usually, the top 1%, 3%, and 10% correct entities are calculated, and the calculation formula is as follows:



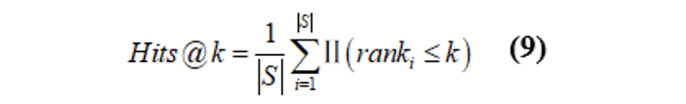



### Evidence-Based Support

The interpretability of prediction results is an important indicator determining the practicality of the machine learning–based model [48]. Although current KG embedding methods can be used to directly predict outcomes, interpretable reasoning is a major challenge owing to the lack of intermediate knowledge in the prediction process [49]. The quantity and quality of the knowledge source can be considered as important factors in assessing the credibility of knowledge in the biomedical field [21]. In this study, we provided an evidence scoring and backtracking mechanism to provide interpretability for the prediction results of the CovKG.

For the entity pair <*e_i_*, *e_j_*>, there may be multiple reachable paths between them, denoted as *p_i_*_,_*_j_*. In these paths, *e_i_* and *e_j_* may be directly connected, that is, *p_i_*_,_*_j_*=<*e_i_*,*r_i_*_,_*_j_*,*e_j_*>. They may also be connected by multiple hops through other nodes connected to them, that is, *p_i_*_,_*_j_*=<*e_i_*,*r_i_*_,_*_x_*,*e_x_*,*…*,*e_y_*,*r_y_*_,_*_j_*,*e_j_*>. The biomedical literature supporting the existence of these paths is the evidence. The evidence number for the path *p_k_* is denoted as *Count*(*p_k_*). The evidence score of the predicted link of the entity pair <*e_i_*, *e_j_*> is the sum of the evidence number in all reachable paths, and the formula is as follows:



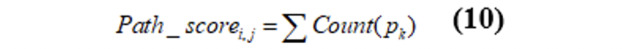



Additionally, the source information (eg, PMID, TiID, and AbID) of the biomedical entity involved in the literature has been considered during the design process of the semantic structure of PubMedAnn. Therefore, for each triple predicted by the KG embedding model, it can be traced back to its original source, including its specific literature, abstract, and title. In the example shown in Figure 2, an explanation path “Epithelial Cells-LOCATION_OF-Defensins-ISA-Immunologic Factors” can be generated for the predicted result about “Epithelial Cells” and “Immunologic Factors” in the abstract of PMID 34206990.

## Results

### CovKG Statistics

The CovKG we constructed contains 17,369,620 triples. Among them, PubMedAnn constructed from biomedical literature contains 641,195 triples, covering 13,065 CUIs, 209 semantic types, and 97 semantic relations of the UMLS. Table 1 shows the high-frequency triples related to diseases, drugs, and genes in PubMedAnn.

Through multi-source knowledge integration, 475 drugs and 262 targets in PubMedAnn were mapped to DrugBank and GO, respectively. The top 10 high-frequency drugs in PubMedAnn were “hydroxychloroquine,” “chloroquine,” “azithromycin,” “dexamethasone,” “ribavirin,” “colchicine,” “ergocalciferol,” “ivermectin,” “methylprednisolone,” and “ritonavir.” The top 10 high-frequency targets in PubMedAnn were “angiotensin converting enzyme 2,” “M Protein, multiple myeloma,” “inflammatory response,” “vitronectin, human,” “endopeptidases,” “measles virus nucleoprotein,” “cytokine,” “peptides,” “TMPRSS2 gene,” and “glycoproteins.”

**Table 1:** Top 10 triples in PubMedAnn (eliminated generic biomedical semantic types).

Head entity	Relation	Tail entity	Count
Coronavirus infections	CAUSES	Disease	680
Coronavirus infections	AFFECTS	Severe acute respiratory syndrome	322
M Protein, multiple myeloma	PART_OF	SARS coronavirus	164
Hydroxychloroquine	TREATS	Patients	138
Assay	METHOD_OF	Detection	131
Measles virus nucleoprotein	PART_OF	SARS coronavirus	129
Fever	COEXISTS_WITH	Symptoms	97
Hydroxychloroquine	INTERACTS_WITH	Chloroquine	92
ACE2 gene	PART_OF	Homo sapiens	81
Serum	LOCATION_OF	Antibodies	80

### Unrecorded Drug Mechanism of Action Discovery

The constructed CovKG was used to explore the mechanisms of drug action that have not been recorded in DrugBank. Here, “the mechanism of drug action” is a broad semantic type, which refers to the physiological process of drug action on targets or hosts, such as “gene or genome,” “genetic function,” “molecular function,” “biologic function,” etc.

Owing to KG technologies, the SPARQL query and logical reasoning statements related to the semantic triple <subject, predicate, object> can be constructed to obtain the relations between drugs and targets in PubMedAnn, where the subject (ie, head entity) is the drug name in DrugBank and the object (ie, tail entity) is the gene name in GO. The results were compared with all triples in DrugBank whose semantic type is “drug-relation-gene,” and a total of 41 mechanisms of action for drug repurposing that have not been recorded in DrugBank were found (Table 2).

**Table 2:** Unrecorded mechanisms of action for drug repurposing.

Head entity	Relation	Tail entity
Amiodarone	AFFECTS	Late endosome
Lopinavir	AFFECTS	Phosphatidylinositol 3-kinase signaling
Ergocalciferol	AFFECTS	Inflammatory response
Allopurinol	AFFECTS	Peptidase activity
Naproxen	AFFECTS	Peptidase activity
Methylprednisolone	AFFECTS	RNA replication
Colchicine	AFFECTS	Inflammatory response
Teicoplanin	AFFECTS	Phosphatidylinositol 3-kinase signaling
Colchicine	AFFECTS	Neutrophil degranulation
Amantadine	AFFECTS	Ion channel activity
Platelet activating factor	AUGMENTS	Mast cell activation
Selenium	AUGMENTS	RNA replication
Doxorubicin	CAUSES	Neutrophil apoptotic process
Amantadine	DISRUPTS	Channel activity
Miglustat	DISRUPTS	N-glycan processing
Serotonin	DISRUPTS	Inflammatory response
Luteolin	DISRUPTS	Cell activation
Ritonavir	DISRUPTS	Exoribonuclease activity
Chlorpromazine	DISRUPTS	Clathrin-dependent endocytosis
Roxadustat	DISRUPTS	RNA replication
Arginine	DISRUPTS	P-body
Fenretinide	DISRUPTS	Syncytium formation by plasma membrane fusion
Doxorubicin	DISRUPTS	Inflammatory response
Doxycycline	DISRUPTS	Transduction
Chloroquine	DISRUPTS	Endolysosome
Niclosamide	DISRUPTS	Virus maturation
Ivermectin	DISRUPTS	Nucleocytoplasmic transport
Metformin	DISRUPTS	Monocyte activation
Metformin	DISRUPTS	Inflammatory response
Fluvoxamine	DISRUPTS	Cytokine production
Rimantadine	NEG_AFFECTS	Ion channel activity
Amiloride	NEG_DISRUPTS	Ion channel activity
Ergocalciferol	PREDISPOSES	Inflammatory response
Bradykinin	PREVENTS	Inflammatory response
Nicotine	TREATS	Inflammatory response
GTS-21	TREATS	Inflammatory response
Viral membrane	LOCATION_OF	Alanine
Late endosome	LOCATION_OF	Hydroxychloroquine
Food vacuole	LOCATION_OF	Chloroquine
Host cell membrane	LOCATION_OF	Cholesterol
Cytokine production	PRODUCES	Nitric oxide

### KG Embedding

#### Performance Comparison

The performances of the 6 state-of-the-art KG embedding models are shown in Table 3. Higher scores of MRR and Hits@*k* (*k*=1, 3, and 10) are associated with better predictive performance of the model. The results showed that TransR performed the best on all metrics. The optimal TransR configuration was achieved with *emb_size*=400 hidden dimensions, learning rate *lr*=0.25, regularization coefficient *λ*=1e-09, *max_train_step*=6000, and *batch_size*=1000.

**Table 3:** Performance of the knowledge graph embedding models.

Model	MRR^a^	Hits@1	Hits@3	Hits@10
TransE	0.1987	0.1280	0.2182	0.3014
TransR	0.2510^b^	0.2011^b^	0.2967^b^	0.3505^b^
RotatE	0.2016	0.1475	0.2307	0.2980
RESCAL	0.2209	0.1897	0.2407	0.2799
DistMult	0.1302	0.0985	0.1390	0.2210
ComplEx	0.1497	0.1209	0.1518	0.2269

^a^MRR: mean reciprocal rank.

^b^Best results.

#### Potential Target Discovery

To predict potential targets, 3 data sets were constructed for entities and their relations, namely, the known entity set *s_e_*, the candidate entity set *s_e′_*, and the candidate relation set *s_r_*. We extended the term “coronavirus” to construct the known entity set, and 39 terms were acquired by retrieving in the CovKG, involving coronavirus name, virus variant name, and abbreviated name. Correspondingly, all other terms were regarded as candidate entities. The 361 relations contained in the CovKG were defined as the relation candidate set.

The KG embedding models described in the Methods section were used to predict the potential targets related to coronaviruses. The entities in the known entity set *s_e_* were taken as the head entities or tail entities. The link prediction results for potential target discovery are presented in Table 4.

To assess the reliability of the predictions and provide the interpretability of the results, the explanation paths were extracted. Each path can be regarded as a combination of triples, and the evidence score of each triple can be calculated by the amount of evidence reported in the literature. The sum of the scores of all triples is the credibility of the path. For example, in Table 4, the explanation path “SARS coronavirus RNA - AUGMENTS - Membrane - PART_OF - Virus - LOCATION_OF - CD69 protein, human” can be split into “SARS coronavirus RNA - AUGMENTS - Membrane,” “Membrane - PART_OF - Virus,” and “Virus - LOCATION_OF - CD69 protein, human.” Since the evidence scores for each triple were 1, 2, and 3, respectively, the path had a confidence score of 6.

Furthermore, to investigate the molecular features of potential targets, the public gene expression data for transcriptome profiling were obtained from The Cancer Genome Atlas (TCGA) database. There were 59 normal lung tissues from TCGA-LUAD. We downloaded the RNA sequencing data (fragments per kilobase of transcript per million mapped reads [FPKM] value) from TCGA. All of the RNA sequencing data were adjusted for background adjustment and quantile normalization with a robust multiarray averaging method in the “affy” and “simpleaffy” packages.

**Figure 6:**
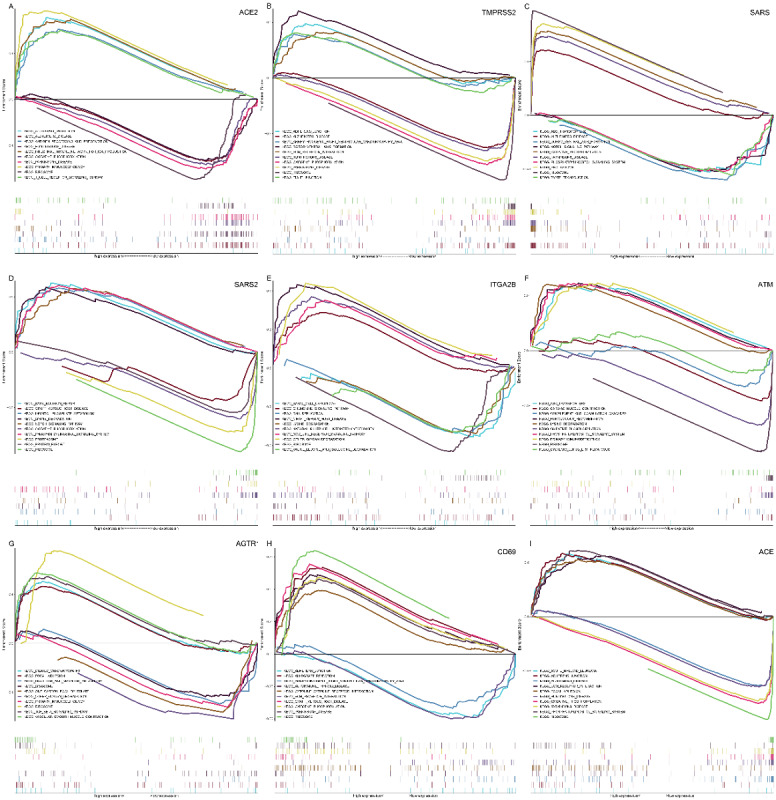
Gene set enrichment analysis of the discovered targets of coronaviruses. (A) ACE2; (B) TMPRSS2; (C) SARS; (D) SARS2; (E) ITGA2B; (F) ATM; (G) AGTR1; (H) CD69; (I) ACE. The X-axis indicates the level of gene expression, and the Y-axis indicates the enrichment score. ACE: angiotensin I converting enzyme; ACE2: angiotensin converting enzyme 2; AGTR1: angiotensin II receptor type 1; ATM: ATM serine/threonine kinase; CD69: cluster of differentiation 69; ITGA2B: integrin subunit alpha 2b; TMPRSS2: transmembrane serine protease 2. For a higher-resolution version of this figure, see Multimedia Appendix 2.

We performed gene set enrichment analysis (GSEA) with GSEA 4.3.2 (UC San Diego and Broad Institute). The gene set of “C2: curated gene sets [including Kyoto Encyclopedia of Genes and Genomes (KEGG)]” was downloaded from the MSigDB database. We set the nominal (NOM) at *P*<.05 and the enrichment score (ES) at >0.2 or <−0.2 for statistical significance to identify the difference in the biological process, and the 5 sets with the highest ESs are shown in Figure 6. According to the KEGG collection defined by MSigDB, some potential targets were related, including the adherens junction, primary immunodeficiency, and oxidative phosphorylation. The raw data of differential gene expression are shown in Multimedia Appendix 1.

**Table 4:** Link prediction results for potential target discovery.

Link prediction result	Explanation path^a^	Evidence
SARS coronavirus RNA - AFFECTS - CD69 protein, human	SARS coronavirus RNA - AUGMENTS (1) - Membrane - PART_OF (2) - Virus - LOCATION_OF (3) - CD69 protein, human	PMID^b^ 18451981, 34323931, and 33862647
3C - like protease, SARS coronavirus - INTERACTS_WITH - Endopeptidases	3C - like protease, SARS coronavirus - compared_with (1) - Hydroxychloroquine - COEXISTS_WITH (61) - Pharmaceutical Preparations - COEXISTS_WITH (22) - Endopeptidases	PMID 32720578, 33305554, and 15890949
M protein, Coronavirus - COEXISTS_WITH - M Protein, multiple myeloma	M protein, Coronavirus - CAUSES (1) - Apoptosis - AFFECTS (4) - Severe Acute Respiratory Syndrome - PRODUCES (22) - M Protein, multiple myeloma	PMID 16797548, 12919893, and 16893997
SARS2 gene - USES -TMPRSS2 gene	SARS2 gene - CAUSES (1) - Disease - AFFECTS (357) - Patients - LOCATION_OF (3) - TMPRSS2 gene	PMID 32703328, 15271120, and 33796097
angiotensin converting enzyme 2 - INTERACTS_WITH - M Protein, multiple myeloma	angiotensin converting enzyme 2 - LOCATION_OF (5) - Mutation - AFFECTS (1) - RNA Recognition Motif - COEXISTS_WITH (10) - M Protein, multiple myeloma	PMID 22291007, 34648284, and 26038424
ACE2 protein, human - INHIBITS - anti - IgG	ACE2 protein, human - CAUSES (1) - Severe disorder - PROCESS_OF (82) - Patients - LOCATION_OF (7) - anti - IgG	PMID 34185681, 34117116, and 15631740
Human coronavirus 229E - INTERACTS_WITH - M Protein, multiple myeloma	Human coronavirus 229E - LOCATION_OF (1) - Replicon - PART_OF (7) - SARS coronavirus - LOCATION_OF (164) - M Protein, multiple myeloma	PMID 15890949, 16928748, and 15840526
nucleocapsid protein, Coronavirus - STIMULATES - TMPRSS2 gene	nucleocapsid protein, Coronavirus - AFFECTS (3) - Cells - LOCATION_OF (9) - ACE2 gene - COEXISTS_WITH (24) - TMPRSS2 gene	PMID 16734668, 32564046, and 33243116
Ataxia Telangiectasia Mutated Proteins - INTERACTS_WITH - ACE2 protein, human	Ataxia Telangiectasia Mutated Proteins - INTERACTS_WITH (1) - ACE2 gene - LOCATION_OF (2) - receptor expression - PROCESS_OF (1) - ACE2 protein, human	PMID 33728680, 32980345, and 33617712
AGTR1 gene - STIMULATES - angiotensin converting enzyme 2	AGTR1 gene - ASSOCIATED_WITH (1) - Infiltration - CAUSES (3) - Severe Acute Respiratory Syndrome - PRODUCES (28) - angiotensin converting enzyme 2	PMID 34671200, 15837019, and 34755492
stinging nettle lectin -AFFECTS - Coronavirus antibody	stinging nettle lectin - DISRUPTS (1) - Severe Acute Respiratory Syndrome - AFFECTS (1324) - Patients - LOCATION_OF (1) - Coronavirus antibody	PMID 21338626, 15640317, and 14574997
Interferon Type I - INTERACTS_WITH - Spike Glycoprotein, Coronavirus	Interferon Type I - COEXISTS_WITH (4) - Genes - INTERACTS_WITH - Escherichia coli (4) - LOCATION_OF (3) - Spike Glycoprotein, Coronavirus	PMID 34341659, 18050746, and 16273643

^a^Given the large amount of evidence, only 1 explanation path for each link prediction result is listed. “()” indicates the confidence score of each triple.

^b^PMID: PubMed ID.

#### Drug Repurposing

By querying DrugBank, the recorded drugs having therapeutic effects on “SARS-CoV-2,” “MERS-CoV,” and “SARS-CoV” were “ritonavir,” “chloroquine,” “darunavir,” “lopinavir,” “elbasvir,” “umifenovir,” “remdesivir,” “human interferon beta,” “TMC-310911,” “N4-hydroxycytidine,” and “EIDD-2801.” To explore the potential anticoronavirus drugs, 6 KG embedding models were used to find drugs that are the most similar to the above 11 drugs.

**Figure 7:**
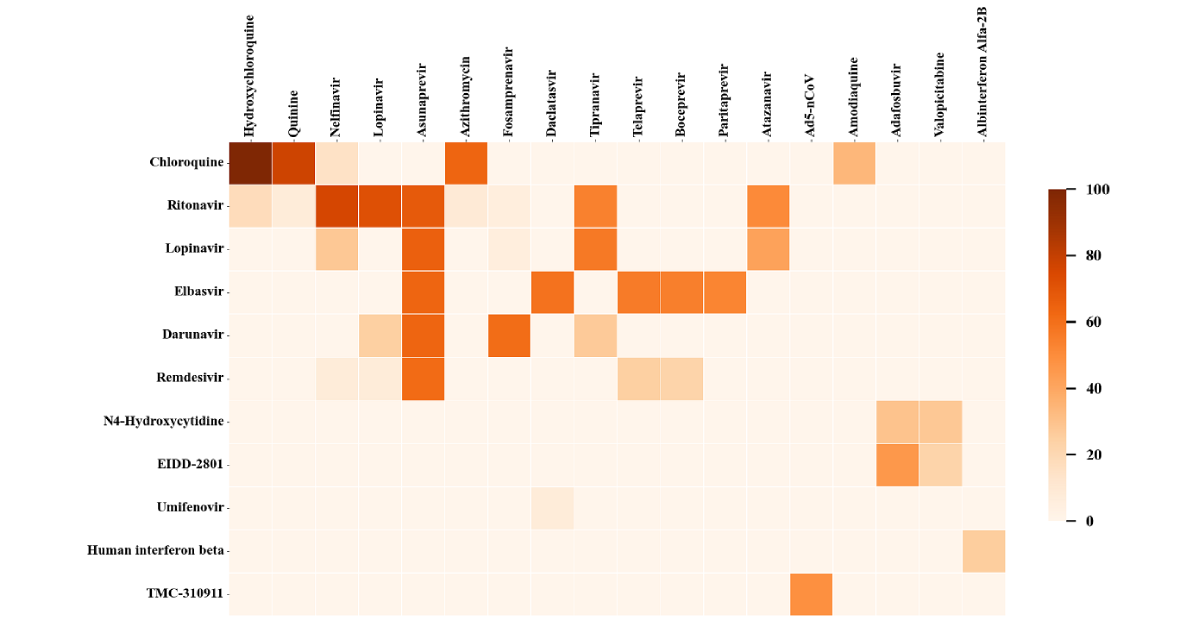
Heatmap depicting the strength of the association between the drugs in DrugBank (vertical axis) and the prediction results (horizontal axis), using the similarity calculation method. A darker color is associated with greater similarity between the corresponding 2 drugs.

Figure 7 shows the prediction results visualized through a heat map. It can be seen that all models consistently predicted the highest similarity between “chloroquine” and “hydroxychloroquine.”

To generate explanations for the predicted results, the predicted drugs and coronaviruses were formed into a “query pair” to obtain explanation paths, as shown in Table 5.

**Table 5:** Potential drug explanation paths with evidence-based support.

Query pair	Explanation path^a^	Evidence
Hydroxychloroquine - Coronavirus Infections	Hydroxychloroquine - TREATS (24) - Disease - CAUSES (202) - Severe Acute Respiratory Syndrome - COEXISTS_WITH (322) - Coronavirus Infections	PMID^b^ 34919258, 18380809, and 17942563
Quinine - Coronavirus Infections	Quinine - AFFECTS (3) - Virus Diseases - AFFECTS - Disease - AFFECTS (680) - Coronavirus Infections	PMID 33918670, 15780893, and 14726162
Nelfinavir - Human coronavirus	Nelfinavir - AFFECTS (1) - Membrane Fusion - AFFECTS (8) - Glycoproteins - PART_OF (1) - Human coronavirus	PMID 32374457, 15262502, and 34716096
Lopinavir/Ritonavir - Middle East Respiratory Syndrome Coronavirus	lopinavir/Ritonavir - COEXISTS_WITH (8) - arbidol - TREATS (2) - Pneumonia - COEXISTS_WITH (3) - Middle East Respiratory Syndrome Coronavirus	PMID 34269012, 34763510, and 28616501
Amodiaquine - SARS coronavirus	Amodiaquine - ISA (2) - Antimalarials - AUGMENTS (1) - Apoptosis - AFFECTS (17) - SARS coronavirus	PMID 33941899, 14975502, and 16107218
Daclatasvir - Coronavirus Infections	Daclatasvir - ISA (2) - Pharmaceutical Preparations - AFFECTS (126) - Disease - AFFECTS (680) - Coronavirus Infections	PMID 32720571, 23434688, and 15690493
Tipranavir - Coronavirus Infections	Tipranavir - INTERACTS_WITH (1) - Enzymes - AFFECTS (1) - Malignant Neoplasms - COEXISTS_WITH (10) - Coronavirus Infections	PMID 32752938, 33768439, and 32845538
Telaprevir - Coronavirus OC43, Human	Telaprevir - ISA (2) - Pharmaceutical Preparations - AFFECTS (126) - Disease - COEXISTS_WITH (1) - Coronavirus OC43, Human	PMID 33360831, 34427335, and 23532101
Boceprevir - Coronavirus Infections	Boceprevir - ISA (2) - Pharmaceutical Preparations - AFFECTS (126) - Disease - AFFECTS (680) - Coronavirus Infections	PMID 33373194, 23434688, and 15690493
Paritaprevir - Coronavirus Infections	Paritaprevir - ISA (2) - Pharmaceutical Preparations - STIMULATES (5) - Interferons - DISRUPTS (1) - Coronavirus Infections	PMID 33984267, 15739619, and 25093995
Atazanavir - Coronavirus Infections	Atazanavir - COEXISTS_WITH (1) - lopinavir - ISA (2) - Antiviral Agents - TREATS (13) - Coronavirus Infections	PMID 33156364, 33504301, and 32335561
Azithromycin - Spike Glycoprotein, Coronavirus	Azithromycin - COEXISTS_WITH (54) - Hydroxychloroquine - INTERACTS_WITH (1) - Spike Glycoprotein, Coronavirus	PMID 32923151 and 33525415
Tipranavir - Genus: Coronavirus	Tipranavir - INTERACTS_WITH (1) - Enzymes - PART_OF (1) - Genus: Coronavirus	PMID 32752938 and 33530371

^a^Given the large amount of evidence, only 1 explanation path for each link prediction result is listed. “()” indicates the confidence score of each triple.

^b^PMID: PubMed ID.

## Discussion

### Model Performance

The evidence-based analysis of the results showed that all models adopted in this study could identify drugs and targets for coronaviruses with KGs. Among them, TransR performed the best in translational models and RESCAL performed the best in semantic matching models. TransR optimized the projection of complex relations and could well represent the semantic connection between entities and relations. However, owing to the large number of model parameters, it took the longest training time compared with other models. RESCAL took the shortest training time and showed the second best performance on our data set. Although DistMult and ComplEx were improved models of RESCAL, they did not fit our data well from the experimental results. TransE, RotatE, DistMult, and ComplEx performed poorly on MRR and Hits@k, but these models were still able to predict most drugs and targets.

### Knowledge Discovery

#### Unrecorded Mechanisms of Drug Action

We constructed CovKG by integrating multi-source knowledge in a semantic way and identified 41 mechanisms of drug action not recorded in DrugBank. We have discussed 3 mechanisms of drug action in Table 2.

##### Colchicine-AFFECTS-Inflammatory Response

Colchicine has good therapeutic effects on myocardial infarction, atherosclerosis, gout, etc. It can interfere with multiple inflammatory pathways and attenuate the inflammatory response through recruitment of neutrophils [50]. Some studies have shown that colchicine may reduce the hospitalization rate, the death rate, and the incidence of serious adverse events in patients with COVID-19 [51].

##### Niclosamide-DISRUPTS-Virus Maturation

The main antiviral mechanism of niclosamide (NCL) involves neutralizing endosomal pH and inhibiting viral protein maturation. NCL exerts its anti-SARS-CoV-2 effect by blocking the viral life cycle and inducing cytopathic effects [52]. In addition, studies have shown that NCL has a significant inhibitory effect on SARS-CoV [53]. Immunoblotting results showed that the synthesis of viral antigen was completely inhibited when NCL was used at a concentration of 1.56 μm. NCL has been shown to have significant anti-inflammatory and antiviral effects [54].

##### Methylprednisolone-AFFECTS-RNA Replication

A survival analysis of critical patients with COVID-19 treated with methylprednisolone has been performed. Analysis of clinical outcomes and laboratory results revealed significant differences in recovery time and transfer to intensive care between the experimental and control groups [55]. Methylprednisolone may be an effective treatment for critical patients with COVID-19 [56]. Lung-protective ventilation and methylprednisolone infusion therapy have also been certified to be useful in the management of acute respiratory distress syndrome in patients with severe MERS and SARS [57]. However, a retrospective study of patients with SARS showed an overall increase in phosphatidylinositol and lysophosphatidylinositol levels in recovered patients who had received methylprednisolone. High-dose methylprednisolone pulses may cause long-term systemic injury associated with altered serum metabolism [58].

#### Potential Therapeutic Drugs

The Anatomical Therapeutic Chemical (ATC) classification system, providing the therapeutic and pharmacological classification of drugs, is the official drug classification system of the World Health Organization [59]. Here, 11 coronavirus drugs recorded in DrugBank and 18 potential therapeutic drugs predicted by the KG embedding models have been discussed combined with the ATC system. In the ATC system, the predicted drugs are concentrated in 4 categories, namely, “P01B: ANTIMALARIALS,” “J05A: DIRECT ACTING ANTIVIRALS,” “J01F: MACROLIDES, LINCOSAMIDES AND STREPTOGRAMINS,” and “L03A: IMMUNOSTIMULANTS.” Most of the drugs are “ANTIMALARIALS” or “DIRECT ACTING ANTIVIRALS.” Drugs in the “ANTIMALARIALS” category include hydroxychloroquine, quinine, amodiaquine, and chloroquine. Drugs in the “DIRECT ACTING ANTIVIRALS” category include nelfinavir, lopinavir, asunaprevir, fosamprenavir, daclatasvir, tipranavir, telaprevir, boceprevir, ombitasvir, paritaprevir, atazanavir, ritonavir, elbasvir, darunavir, remdesivir, and umifenovir.

##### ANTIMALARIALS Category

Hydroxychloroquine, used to treat malaria and some autoimmune disorders, was effective in inhibiting SARS-CoV-1 and SARS-CoV-2 infections in cell culture studies [60]. However, human clinical trials of hydroxychloroquine failed to establish its effectiveness in the treatment of COVID-19, but hydroxychloroquine and chloroquine may easily cause adverse drug reactions [61]. Some recent studies have pointed out that the extracts of quinine and *Artemisia annua* inhibit SARS-CoV-2 infection [62,63]. Further studies would determine in vivo efficacy to assess whether quinine and *A. annua* could provide a cost-effective treatment for SARS-CoV-2 infection [64].

##### DIRECT-ACTING ANTIVIRALS Category

The most concerned drugs in current research are lopinavir, ritonavir, nelfinavir, and darunavir [65]. The combination of lopinavir and ritonavir used to treat and prevent HIV infection has been adopted in hospitals to treat COVID-19 [66]. However, some randomized controlled trials found no evidence that lopinavir and ritonavir are associated with improved mortality or other clinical outcomes [67]. Several studies have shown that the transient expression of the SARS CoV-2 S glycoprotein in Vero cells leads to extensive cell fusion. Nelfinavir mesylate (Viracept) significantly inhibited S-o-mediated cell fusion with complete inhibition at a concentration of 10 μM. In addition, nelfinavir may inhibit S proteolytic processing intracellularly [68]. These results warrant further investigations of the potential of nelfinavir mesylate to inhibit virus spread at early times after the onset of the symptoms of SARS CoV-2 infection.

We also found some drugs and corresponding genes associated with coronavirus infection through link prediction. The RotatE model predicted a high correlation between ivermectin and the coronavirus. By using the evidence-based mechanism, we found a study stating that ivermectin appears to be efficacious in providing clinical benefits in the randomized treatment of asymptomatic SARS-CoV-2–positive subjects, effectively resulting in fewer symptoms, lower viral load, and reduced hospital admissions [69]. Four linkage prediction models predicted that asunaprevir could inhibit *SARS* gene expression. Some studies have found that asunaprevir displays broad-spectrum antiviral activity. Asunaprevir markedly inhibited SARS-CoV-2–induced cytopathic effects in Vero E6 cells. Both the RNA and protein levels of SARS-CoV-2 were significantly reduced after treatment with asunaprevir [70]. The TransE model predicted that resveratrol has an effect on the angiotensin converting enzyme 2 (*ACE2*) gene. Resveratrol has been shown to mitigate the major pathways involved in the pathogenesis of SARS-CoV-2, including modulation of the renin-angiotensin system and expression of ACE2, stimulation of the immune system, and downregulation of proinflammatory cytokine release [71]. The TransE model predicted that tylophorine could treat MERS-CoV. Several studies suggested that tylophorine-based compounds exert broad spectrum and potent inhibitory effects against coronaviruses. In previous studies, combination treatment, wherein a tylophorine-based compound targeted transmissible gastroenteritis virus and a Janus kinase 2 (JAK2) inhibitor blocked the alternative dominant nuclear factor κB (NF-κB) activation mediated by JAK2, was more effective and comprehensive than either treatment alone, and constituted a feasible approach for the treatment of SARS-CoV and MERS-CoV infections [72]. After the outbreak of COVID-19, 3 tylophorine-based compounds were further examined for their antiviral activities on SARS-CoV-2, with inhibition of SARS-CoV-2 at EC50 values of up to 2.5-14 nM [73].

#### Potential Therapeutic Targets

**Figure 8:**
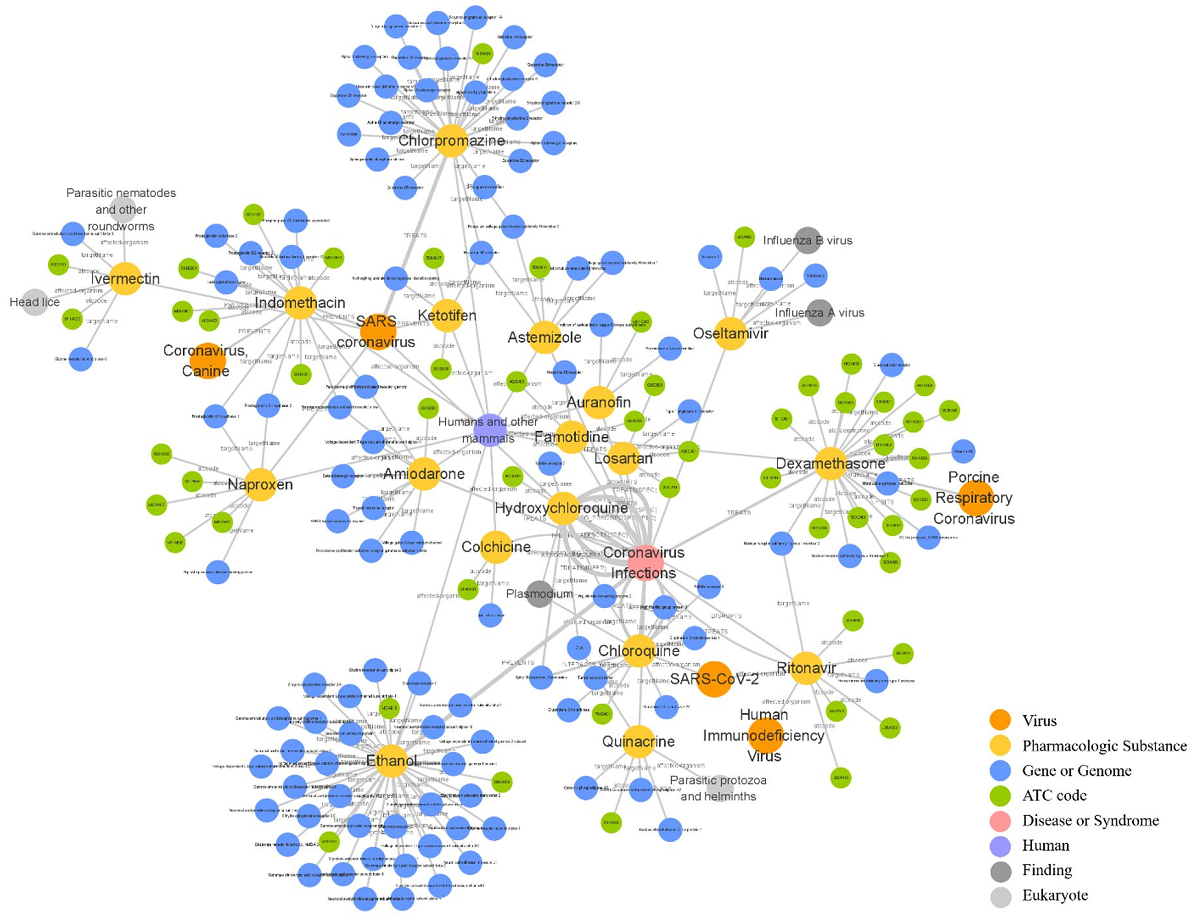
Potential target discovery and drug repurposing for coronaviruses with knowledge graphs. The thickness of the edge between 2 entities indicates the credibility of the triple.

Based on the link prediction models, 33 potential targets of coronavirus infection were found. Here, we have discussed 3 common targets in model predictions. Figure 8 visually shows the viruses, drugs, targets, and their interactions of interest in this study.

##### ACE2

The key link between the renin-angiotensin system and COVID-19 is ACE2, which increases the tissue anti-inflammatory response and has been reported to be the primary host-cell receptor for 3 other coronaviruses, namely, HCoV-NL63, SARS-CoV, and SARS-CoV-2 [74]. The interaction between the SARS-CoV-2 spike-in protein and the human receptor ACE2 is influenced by the disulfide-thiol balance in the host cell, and both proteins contain several cysteine residues. Because of an imbalance between reactive oxygen/nitrogen species and antioxidants, the host cell redox status would be affected [75]. Recent studies have shown that vitamin D supplementation reduces oxidative stress and is protective against viral infections, including COVID-19 [76].

##### TMPRSS2

Transmembrane serine protease 2 (TMPRSS2) efficiently activates the SARS-CoV spike protein [77]. SARS-CoV cellular receptors ACE2 and TMPRSS2 were co-expressed in type II pneumocytes, which are important viral target cells, suggesting that SARS S was cleaved by TMPRSS2 in the lungs of SARS-CoV–infected individuals [78]. TMPRSS2 may contribute to virus transmission and pathogenesis by neutralizing antibodies to reduce virus recognition and activating SARS S for cell-cell and virus-cell fusion [79]. Ribavirin inhibits the TMPRSS2 enzyme, as measured by proteolytic activity, thereby interfering with the expressions of TMPRSS2 and ACE2 [80].

##### M Protein

The M protein in coronaviruses plays an important role in viral assembly by bringing together viral and host factors and making the cell membrane the site for viral replication [81,82]. It has been shown that MERS-CoV and SARS-CoV evade host antiviral responses by using their M proteins to inhibit type I IFN expression at the level of tbk1-dependent phosphorylation and IRF3 activation [83,84]. M protein stabilized B-cell lymphoma 2 (BCL-2) ovarian killer (BOK) by inhibiting its ubiquitination and promoted BOK mitochondria translocation. It activated the BOK-dependent apoptotic pathway and thus exacerbated SARS-CoV-2–associated lung injury in vivo. These findings suggest a proapoptotic role for M protein in SARS-CoV-2 pathogenesis, which may provide potential targets for the treatment of COVID-19 [85,86].

### Limitations and Future Work

In this study, we used an off-the-shelf extraction tool to extract relevant information from biomedical text. Thus, the effectiveness of knowledge discovery depends to a certain extent on the accuracy of the predications automatically extracted by SemRep. The accuracy of SemRep in extracting triple relations in the biomedical literature is about 60% [24]. Although evidence-based mechanisms support checking the accuracy of prediction results, inaccurate data still affect the performance of predicted models. In future work, we will consider annotating part of the biomedical text and training a predicate extraction model based on this corpus to improve the automatic extraction performance. Moreover, the source evidence of biomedical knowledge could be further enriched with fine-grained information. For example, different weights can be assigned according to article types, such as randomized controlled trial, review, and meta-analysis, to provide a better evidence base.

Through KG construction and a series of computational methods proposed in this study, potential targets and candidate drugs related to coronaviruses were found, but further laboratory experiments and clinical assessments are still needed.

### Conclusion

In this study, we proposed a heterogeneous knowledge integration approach for potential target discovery and drug repurposing for coronaviruses, using KGs. Different from existing efforts that mainly focused on the knowledge of COVID-19, we not only expanded the scope of the research object to the entire coronavirus family, but also extracted coronavirus-related knowledge of interest from the huge amount of biomedical literature and realized knowledge integration with 2 authoritative knowledge bases (ie, DrugBank and GO). As all these multi-source biomedical data were associated with each other, it helps to improve the reusability of knowledge. To discover unrecorded mechanisms of drug action as well as potential targets and drug candidates, state-of-the-art KG embedding models were evaluated. We identified 33 potential targets and 18 drug candidates for coronaviruses, and the results were validated and discussed with evidence-based support. The approach proposed here is not specific to coronaviruses and can be extended to other viruses or diseases for potential target discovery and drug repurposing, and the knowledge of PharmGKB, OpenTargets, and other typical databases can be integrated to discover unrevealed underlying biomedical knowledge with the improvement of the reusability of knowledge on a larger scale.

Throughout the epidemiological histories of SARS, MERS, and COVID-19, each outbreak has caused panic among human beings and has had a huge impact on the economy. Coronaviruses are constantly evolving, and it is unknown which new coronavirus will emerge and when the next coronavirus will sweep across the world. KG technologies and novel computational methods are expected to discover the pathogenicity and transmission mechanism of viruses. This study provides a reference for the prevention and treatment of diseases caused by coronaviruses, and the proposed methods and research results are beneficial to promote the repurposing of effective antiviral drugs.

## Appendix

Multimedia Appendix 1 Enrichment analysis of the Kyoto Encyclopedia of Genes and Genomes for potential targets.

Multimedia Appendix 2 Gene set enrichment analysis of the discovered targets of coronaviruses. (A) ACE2; (B) TMPRSS2; (C) SARS; (D) SARS2; (E) ITGA2B; (F) ATM; (G) AGTR1; (H) CD69; (I) ACE. The X-axis indicates the level of gene expression, and the Y-axis indicates the enrichment score. ACE: angiotensin I converting enzyme; ACE2: angiotensin converting enzyme 2; AGTR1: angiotensin II receptor type 1; ATM: ATM serine/threonine kinase; CD69: cluster of differentiation 69; ITGA2B: integrin subunit alpha 2b; TMPRSS2: transmembrane serine protease 2.

## Abbreviations

AbID: abstract ID
ACE2: angiotensin converting enzyme 2
ATC: Anatomical Therapeutic Chemical
BOK: B-cell lymphoma 2 ovarian killer
CovKG: coronavirus knowledge graph
CUI: concept unique identifier
ES: enrichment score
GO: Gene Ontology
GSEA: gene set enrichment analysis
JAK2: Janus kinase 2
KEGG: Kyoto Encyclopedia of Genes and Genomes
KG: knowledge graph
MERS: Middle East respiratory syndrome
MERS-CoV: Middle East respiratory syndrome coronavirus
MRR: mean reciprocal rank
NCL: niclosamide
PMID: PubMed ID
RDF: Resource Description Framework
SARS: severe acute respiratory syndrome
SARS-CoV: severe acute respiratory syndrome coronavirus
TCGA: The Cancer Genome Atlas
TiID: title ID
TMPRSS2: transmembrane serine protease 2
UMLS: Unified Medical Language System

## Data Availability

The code and data are available at [87].
