# Supplementary material for: Potential Target Discovery and Drug Repurposing for Coronaviruses: Study Involving a Knowledge Graph–Based Approach
Source: J Med Internet Res. 2023 Oct 20;25:e45225. doi: 10.2196/45225 (PMC10592722; doi:10.2196/45225)
Supplement: Multimedia Appendix 1 [file jmir_v25i1e45225_app1.docx]

**Multimedia Appendix 1.** Enrichment analysis of the Kyoto Encyclopedia of Genes and Genomes for potential targets.

| ACE2 |  |  |
| --- | --- | --- |
| NAME | ES | P-value |
| KEGG_PRIMARY_IMMUNODEFICIENCY | 0.770972 | <0.001 |
| KEGG_INTESTINAL_IMMUNE_NETWORK_FOR_IGA_PRODUCTION | 0.698396 | <0.001 |
| KEGG_T_CELL_RECEPTOR_SIGNALING_PATHWAY | 0.60218 | <0.001 |
| KEGG_ALLOGRAFT_REJECTION | 0.712923 | <0.001 |
| KEGG_ANTIGEN_PROCESSING_AND_PRESENTATION | 0.608839 | <0.001 |
| KEGG_RIBOSOME | -0.70054 | <0.001 |
| KEGG_PARKINSONS_DISEASE | -0.60675 | <0.001 |
| KEGG_OXIDATIVE_PHOSPHORYLATION | -0.57062 | <0.001 |
| KEGG_ALZHEIMERS_DISEASE | -0.54152 | <0.001 |
| KEGG_HUNTINGTONS_DISEASE | -0.53134 | <0.001 |
|  |  |  |
| TMPRSS2 |  |  |
| NAME | ES | P-value |
| KEGG_ADHERENS_JUNCTION | 0.488246 | <0.001 |
| KEGG_DORSO_VENTRAL_AXIS_FORMATION | 0.601149 | 0.009 |
| KEGG_TIGHT_JUNCTION | 0.403737 | <0.001 |
| KEGG_ARRHYTHMOGENIC_RIGHT_VENTRICULAR_CARDIOMYOPATHY_ARVC | 0.392556 | 0.036 |
| KEGG_ECM_RECEPTOR_INTERACTION | 0.411209 | 0.008 |
| KEGG_RIBOSOME | -0.92289 | <0.001 |
| KEGG_OXIDATIVE_PHOSPHORYLATION | -0.78406 | <0.001 |
| KEGG_PARKINSONS_DISEASE | -0.75122 | <0.001 |
| KEGG_HUNTINGTONS_DISEASE | -0.65673 | <0.001 |
| KEGG_ALZHEIMERS_DISEASE | -0.63094 | <0.001 |
|  |  |  |
| SARS1 |  |  |
| NAME | ES | P-value |
| KEGG_RIBOSOME | 0.968285 | <0.001 |
| KEGG_OXIDATIVE_PHOSPHORYLATION | 0.782626 | <0.001 |
| KEGG_PARKINSONS_DISEASE | 0.732734 | <0.001 |
| KEGG_PROTEASOME | 0.850487 | <0.001 |
| KEGG_ALZHEIMERS_DISEASE | 0.6072 | <0.001 |
| KEGG_TASTE_TRANSDUCTION | -0.5854 | 0.002 |
| KEGG_DORSO_VENTRAL_AXIS_FORMATION | -0.64126 | 0.007 |
| KEGG_PHOSPHATIDYLINOSITOL_SIGNALING_SYSTEM | -0.53513 | 0.002 |
| KEGG_ABC_TRANSPORTERS | -0.54584 | 0.012 |
| KEGG_NOTCH_SIGNALING_PATHWAY | -0.54008 | 0.013 |
|  |  |  |
| SARS2 |  |  |
| NAME | ES | P-value |
| KEGG_PHOSPHATIDYLINOSITOL_SIGNALING_SYSTEM | 0.603246 | <0.001 |
| KEGG_BASE_EXCISION_REPAIR | 0.626733 | 0.002 |
| KEGG_INOSITOL_PHOSPHATE_METABOLISM | 0.57875 | 0.001 |
| KEGG_LYSINE_DEGRADATION | 0.588268 | 0.003 |
| KEGG_NOTCH_SIGNALING_PATHWAY | 0.577436 | 0.004 |
| KEGG_RIBOSOME | -0.90528 | <0.001 |
| KEGG_PROTEASOME | -0.73568 | <0.001 |
| KEGG_OXIDATIVE_PHOSPHORYLATION | -0.60583 | <0.001 |
| KEGG_PROTEIN_EXPORT | -0.56908 | <0.001 |
| KEGG_GRAFT_VERSUS_HOST_DISEASE | -0.45776 | <0.001 |
|  |  |  |
| ITGA2B |  |  |
| NAME | ES | P-value |
| KEGG_RIBOSOME | -0.65206 | <0.001 |
| KEGG_BASAL_CELL_CARCINOMA | -0.54661 | <0.001 |
| KEGG_LYSINE_DEGRADATION | -0.53011 | <0.001 |
| KEGG_VALINE_LEUCINE_AND_ISOLEUCINE_DEGRADATION | -0.51277 | 0.0149 |
| KEGG_DNA_REPLICATION | -0.52125 | 0.005 |
| KEGG_NATURAL_KILLER_CELL_MEDIATED_CYTOTOXICITY | 0.55427 | <0.001 |
| KEGG_GRAFT_VERSUS_HOST_DISEASE | 0.649061 | <0.001 |
| KEGG_NOD_LIKE_RECEPTOR_SIGNALING_PATHWAY | 0.528379 | <0.001 |
| KEGG_CHEMOKINE_SIGNALING_PATHWAY | 0.434106 | <0.001 |
| KEGG_OTHER_GLYCAN_DEGRADATION | 0.659061 | 0.022151899 |
|  |  |  |
| ATM |  |  |
| NAME | ES | P-value |
| KEGG_CARDIAC_MUSCLE_CONTRACTION | -0.46354 | <0.001 |
| KEGG_SYSTEMIC_LUPUS_ERYTHEMATOSUS | -0.20982 | <0.001 |
| KEGG_OXIDATIVE_PHOSPHORYLATION | -0.67875 | <0.001 |
| KEGG_RIBOSOME | -0.92306 | <0.001 |
| KEGG_COMPLEMENT_AND_COAGULATION_CASCADES | -0.33698 | <0.001 |
| KEGG_PHOSPHATIDYLINOSITOL_SIGNALING_SYSTEM | 0.607915 | <0.001 |
| KEGG_LYSINE_DEGRADATION | 0.600482 | 0.004 |
| KEGG_PRIMARY_IMMUNODEFICIENCY | 0.612421 | 0.017 |
| KEGG_ABC_TRANSPORTERS | 0.585634 | 0.014 |
| KEGG_HOMOLOGOUS_RECOMBINATION | 0.609224 | 0.03 |
|  |  |  |
| AGTR1 |  |  |
| NAME | ES | P-value |
| KEGG_OTHER_GLYCAN_DEGRADATION | -0.72145 | 0.005 |
| KEGG_ONE_CARBON_POOL_BY_FOLATE | -0.64487 | 0.018 |
| KEGG_PRIMARY_IMMUNODEFICIENCY | -0.54944 | 0.012 |
| KEGG_LYSOSOME | -0.44112 | 0.007 |
| KEGG_FRUCTOSE_AND_MANNOSE_METABOLISM | -0.518 | 0.032 |
| KEGG_RIBOSOME | 0.757497 | <0.001 |
| KEGG_VASCULAR_SMOOTH_MUSCLE_CONTRACTION | 0.575647 | <0.001 |
| KEGG_TGF_BETA_SIGNALING_PATHWAY | 0.545437 | <0.001 |
| KEGG_FOCAL_ADHESION | 0.466559 | <0.001 |
| KEGG_DILATED_CARDIOMYOPATHY | 0.504329 | <0.001 |
|  |  |  |
| CD69 |  |  |
| NAME | ES | P-value |
| KEGG_ECM_RECEPTOR_INTERACTION | -0.50742 | 0.001 |
| KEGG_ADHERENS_JUNCTION | -0.50416 | <0.001 |
| KEGG_ARRHYTHMOGENIC_RIGHT_VENTRICULAR_CARDIOMYOPATHY_ARVC | -0.4268 | 0.037 |
| KEGG_RIBOSOME | 0.798034 | <0.001 |
| KEGG_OXIDATIVE_PHOSPHORYLATION | 0.594467 | <0.001 |
| KEGG_PARKINSONS_DISEASE | 0.579369 | <0.001 |
| KEGG_GRAFT_VERSUS_HOST_DISEASE | 0.694562 | <0.001 |
| KEGG_ALLOGRAFT_REJECTION | 0.669805 | <0.001 |
| KEGG_CYTOKINE_CYTOKINE_RECEPTOR_INTERACTION | 0.49171 | <0.001 |
| KEGG_AUTOIMMUNE_THYROID_DISEASE | 0.612635 | <0.001 |
|  |  |  |
| ACE |  |  |
| NAME | ES | P-value |
| KEGG_PHOSPHATIDYLINOSITOL_SIGNALING_SYSTEM | 0.615199 | <0.001 |
| KEGG_FOCAL_ADHESION | 0.530289 | <0.001 |
| KEGG_ADHERENS_JUNCTION | 0.585654 | <0.001 |
| KEGG_ECM_RECEPTOR_INTERACTION | 0.564557 | <0.001 |
| KEGG_ACUTE_MYELOID_LEUKEMIA | 0.582122 | 0.001 |
| KEGG_RIBOSOME | -0.95841 | <0.001 |
| KEGG_OXIDATIVE_PHOSPHORYLATION | -0.80802 | <0.001 |
| KEGG_PARKINSONS_DISEASE | -0.78605 | <0.001 |
| KEGG_HUNTINGTONS_DISEASE | -0.6547 | <0.001 |
| KEGG_ALZHEIMERS_DISEASE | -0.64663 | <0.001 |

ES: Enrichment score; KEGG: Kyoto Encyclopedia of Genes and Genomes.
